# An Audit Cycle of Gynecological History Documentation in Emergency Surgical Admissions of Female Patients of Childbearing Age Presenting with Acute Abdominal Pain at a District General Hospital

**DOI:** 10.7759/cureus.76945

**Published:** 2025-01-05

**Authors:** Asher Siddiqui, Zohaib Jamal, Nowera Zafar, Muhammad Ijlal Haider, Naqqash Adnan, Zeeshan Khawaja, Imran Alam

**Affiliations:** 1 Department of Surgery, Wrightington, Wigan and Leigh NHS Foundation Trust, Wigan, GBR; 2 Department of Radiology, Mersey and West Lancashire Teaching Hospitals NHS Trust, Merseyside, GBR

**Keywords:** childbearing age, contraception, emergency surgery, gynaecological history, last menstrual period, pregnancy status

## Abstract

Background

Ectopic pregnancy (EP) is a significant cause of maternal morbidity and mortality. Accurate and timely diagnosis is crucial, particularly in women of reproductive age presenting with acute abdominal pain. This audit aimed to assess the completeness and accuracy of gynecological history documentation, including pregnancy status, in female patients admitted for emergency surgery due to abdominal pain.

Methods

A retrospective audit was conducted within a single NHS Trust, analyzing the surgical assessment documents of 50 female patients aged 12-50 years admitted for emergency surgery. Data collected included documentation of pregnancy status, gynecological history, last menstrual period, sexual activity, and contraceptive use. A subsequent audit cycle assessed the impact of an educational intervention on documentation practices.

Results

Initial findings revealed significant deficiencies in the documentation of key gynecological parameters. Pregnancy status was documented in only 14% of cases, and contraceptive use in 20%. A substantial proportion of cases lacked documentation of gynecological history 50% and sexual history 56%. An educational intervention resulted in a significant improvement in the documentation of sexual history, contraceptive use, and pregnancy status.

Conclusion

This audit revealed significant deficiencies in the initial gynecological assessment of female patients with acute abdominal pain, particularly regarding the documentation of pregnancy status, menstrual history, and contraceptive use. The study highlights the need for improved clinical practices, including enhanced medical education, standardized assessment protocols, and electronic documentation of pregnancy status. Continued research is crucial to address these deficiencies and optimize patient care within the NHS.

## Introduction

Abdominal pain constitutes a significant proportion of emergency surgical admissions in developed countries, with associated mortality rates ranging from 0.4% to 4.4% [1,2]. Approximately one-third of these admissions are of female patients of reproductive age (FRA). Within this cohort, the possibility of ectopic pregnancy (EP) should be carefully considered. EP poses a significant risk to maternal health, with the potential for both mortality and morbidity. It is imperative to exclude EP in any sexually active woman of reproductive age presenting with acute abdominal pain. Typically rupturing between six and 12 weeks post conception, EP often presents in individuals unaware of their pregnancy status [3]. This condition remains a leading cause of maternal mortality in early pregnancy, with an estimated 16,000 women affected annually in England and Wales [4]. An investigation highlighting the significant risk to women's health posed by delayed diagnosis of EP reveals that NHS data indicates 30 cases of EP were misdiagnosed in 2017-2018, leading to substantial patient harm [5]. Between 1996 and 2006, a concerning 87 fatalities were reported in the United Kindom (UK), directly linked to diagnostic and therapeutic failures in 56 cases [6].

Given the significant morbidity and mortality associated with EP, particularly in cases of delayed diagnosis, it is imperative to diligently exclude this condition in all women of reproductive age presenting with acute abdominal pain. In accordance with current National Institute for Health and Care Excellence (NICE) guidelines, documentation of pregnancy status is mandatory prior to any elective surgical procedure [7]. A comprehensive preoperative assessment should include a detailed medical history, encompassing the date of the last menstrual period, contraceptive use, sexual activity, and other pertinent gynecological factors. A positive urine or serum beta-human chorionic gonadotropin (β-hCG) test is essential to confirm pregnancy, although it is important to recognize that a negative test does not definitively exclude the possibility of EP, especially in early gestation [8]. This underscores the importance of a comprehensive medical history encompassing the date of the last menstrual period, contraceptive use, sexual activity, and other relevant gynecological factors.

The aims of this study were to assess the completeness and accuracy of documentation of pregnancy status, gynecological history, last menstrual period, sexual activity, and contraceptive use in the medical records of females of reproductive age admitted for emergency surgery due to abdominal pain, and to determine the frequency of pregnancy testing in this population.

A preliminary abstract of this audit was presented at the West Midlands Surgery Society Conference 2022, held at The Royal Hospital Charles Hastings Way, UK, where it was awarded a runner-up prize. 

## Materials and methods

This quality improvement audit was undertaken within the surgical department of Royal Albert Edward Infirmary, a district general hospital in Wigan, UK. Prior to commencing the audit, a formal proposal was submitted to and approved under the code GenSurg/CA/2021-22/09 by the research and audit department of Wrightington, Wigan and Leigh NHS Foundation Trust. The audit periods were: January 1, 2022, to March 31, 2022, for the first cycle and July 1, 2022, to September 30, 2022, for the second cycle

The study population comprised female patients aged 12-50 years who presented to the emergency department with acute abdominal pain and were subsequently admitted under the general surgery service. Patients who had previously been assessed by the gynecology service or undergone a hysterectomy were excluded. 

Data were collected retrospectively from the surgical assessment documents within the Hospital Information System (HIS) software utilized at the Royal Albert Edward Infirmary. Patient information was retrieved using their Medical Record Number (MRN). The surgical assessment document represents the initial clinical record generated by the on-call surgical doctor following the patient's referral from the emergency department, signifying the commencement of surgical involvement in the patient's care.

Data for all patients were subsequently recorded and analyzed using Microsoft Excel (Microsoft Corporation, Redmond, Washington, United States). Fisher exact test was used for the statistical analysis of the data and P < 0.05 was considered statistically significant.

Following the analysis, the results were presented at a routine audit meeting. To disseminate the findings and promote improvement, an email containing a summary of the audit results was sent to all surgical team members. Additionally, discussions were held with junior surgical doctors, emphasizing the significance of accurate documentation and suggesting the use of acronym expansion to enhance clarity and consistency in patient records. A subsequent audit cycle was conducted two months later, involving 50 additional surgical patients, to evaluate the impact of the interventions on compliance with documentation standards. the data were again analysed as mentioned above.

## Results

**Figure 1:**
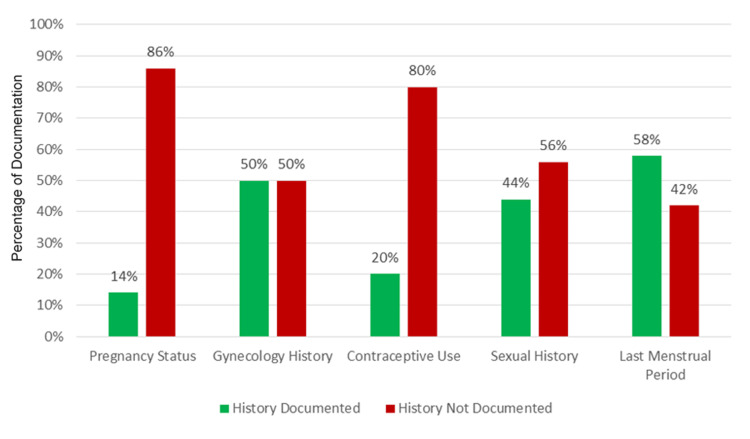
Graph illustrating the documentation compliance rates for the specified audit parameters.

The first half of the audit cycle consisted of assessing the surgical assessment documents of 50 female patients admitted under the surgery. An analysis of surgical assessment documents revealed significant inconsistencies in the documentation of key gynecological history components. Pregnancy status was the least consistently documented element, with only seven (14%) cases including this information. Contraceptive use was also poorly documented, with only 10 (20%) cases providing details. Notably, approximately half of the cases lacked documentation of gynecological history (n=25, 50%) and sexual history (n=22, 44%). The last menstrual period was the most consistently documented factor, appearing in 29 (58%) cases. Additionally, only 17 (34%) patient records documented the results of urine pregnancy tests. These findings are summarized in Figure 1.

**Figure 2:**
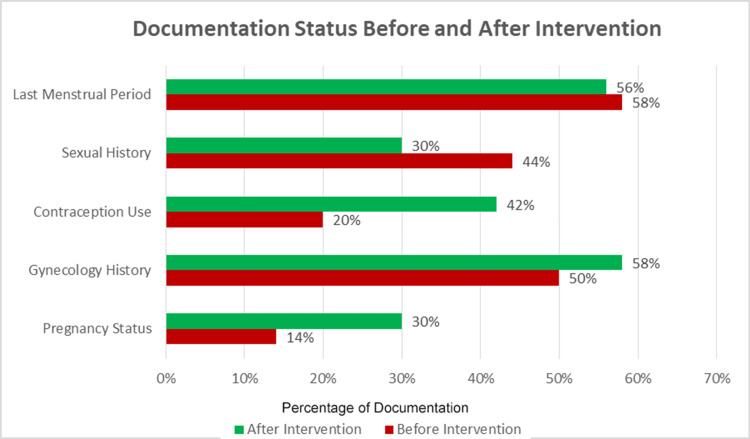
Graph illustrating the pre- and post-intervention documentation status of the audit parameters

**Figure 3:**
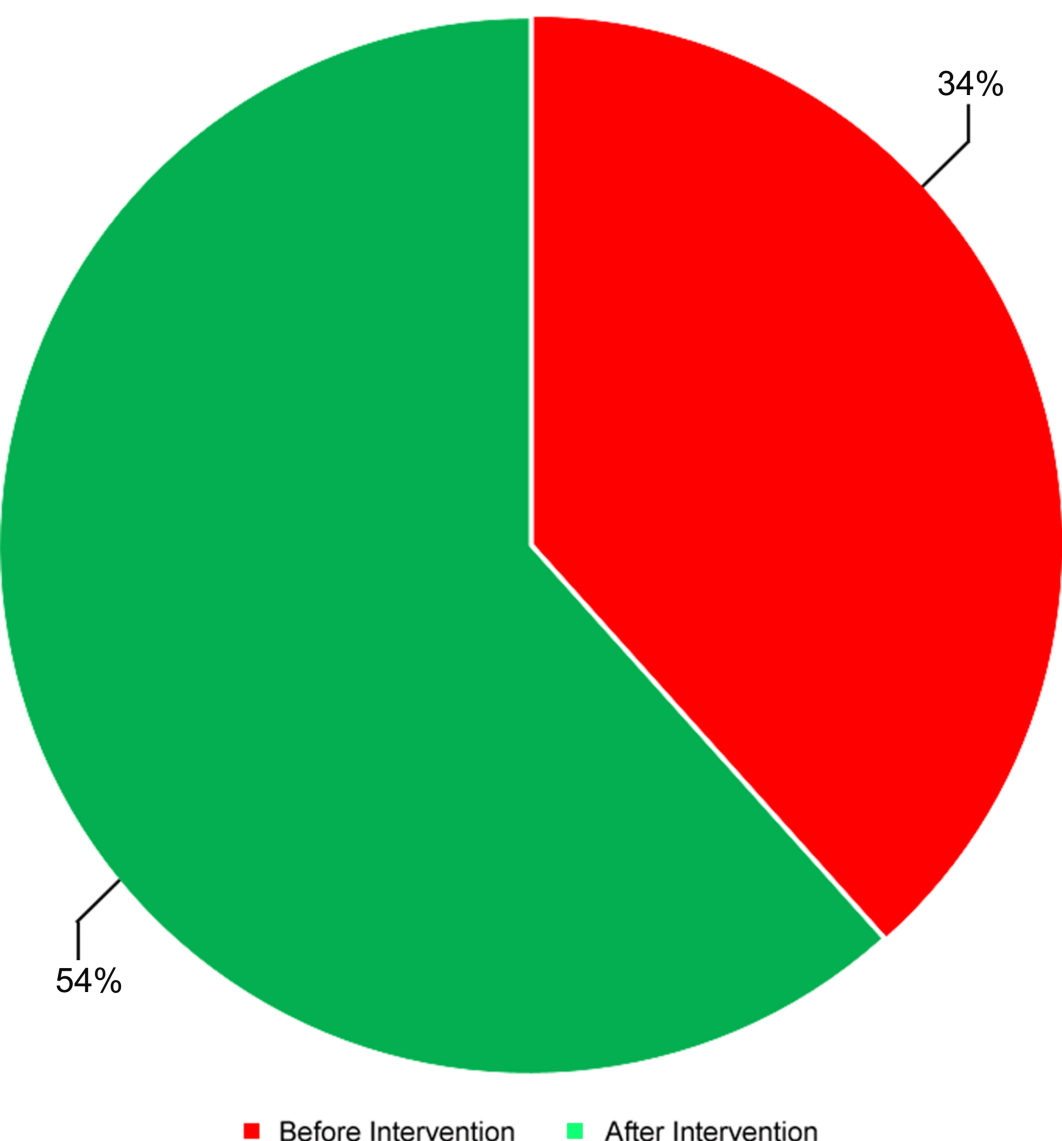
Pie chart illustrating the documentation of urine pregnancy test results pre- and post-intervention

Following the presentation of these findings at a local departmental audit meeting, a second round of audit was conducted two months later to assess compliance with the recommendations again. Notably, a substantial improvement was observed in the documentation of gynecological history, contraceptive use, and pregnancy status post-intervention. The documentation of the last menstrual period showed no substantial change. However, the documentation of sexual history demonstrated a decrease of approximately one-third, as illustrated in Figure 2. On the other hand, the documentation of urine pregnancy test results increased from 17 (34%) to 27 (54%) following the intervention (Figure 3). Statistical analysis using Fisher's Exact Test demonstrated statistically significant improvements in the documentation of all parameters except for the last menstrual period as summarized in Table 1.

**Table 1:** Contingency table of the study parameters and Fisher's exact test p-values

Parameter	Before Intervention		After Intervention		
	Documented	Not Documented	Documented	Not Documented	p-value
Pregnancy Status	7	43	15	35	0.0004
Contraceptive Use	10	40	21	29	0.000000154
Gynecological History	25	25	29	21	0.0259
Sexual History	22	28	15	35	0.0023
Last Menstrual Period	29	21	28	22	0.88
Urine Pregnancy Test Results	17	33	27	23	0.00012

## Discussion

This audit of female patients of childbearing age presenting with emergency abdominal pain in our institution revealed a concerning deficiency in the initial assessment and documentation of key gynecological parameters, including pregnancy status, menstrual history, and contraceptive use. The findings of this audit are consistent with those of previous studies, including an audit by Powell-Bowns et al. [4] and a national multicenter audit conducted by Wilson et al. [9], both of which also demonstrated a substantial lack of appropriate documentation of these crucial historical elements.

The current study unequivocally highlights a significant need for enhanced gynecological assessment within both elective and emergency surgical patient populations. In patients presenting with abdominal pain, the exclusion of EP is paramount. While a negative pregnancy test remains the definitive diagnostic tool, meticulous inquiry into menstrual history, contraceptive use, and sexual activity is crucial for identifying potential risk factors. Reitz et al. reported a case of a false negative pregnancy test in a female patient with multiple gestation intrauterine pregnancy where history taking played a key role in determining the possibility of pregnancy [10]. Moreover, careful consideration of pregnancy status, alongside potential contributing factors, is essential prior to exposure to ionizing radiation or the administration of general anesthesia in emergency surgical settings. A study by Mole found that a major factor in the decrease in childhood cancer deaths was concerted action initiated in 1956 to reduce radiation exposure of fetal gonads for fear of genetic hazards [11]. The National Patient Safety Agency documented 42 instances (between 2003 and 2009) of NHS patients undergoing elective procedures without documented evidence of a negative hCG result, who were subsequently found to be pregnant [12]. Three of these cases resulted in spontaneous abortion. This suggests that the true incidence of such occurrences in emergency surgical patients may be underreported. 

Addressing this deficiency requires a multi-faceted approach. This includes targeted medical education at both undergraduate and postgraduate levels, complemented by the implementation of a standardized gynecological assessment questionnaire specifically designed for female patients of reproductive age. This readily accessible questionnaire, integrated within surgical admission documentation, will serve as a valuable mnemonic device, minimizing the omission of critical information during initial patient evaluations. This approach aligns with the multifaceted strategy proposed by Wilson et al. to address this deficiency, emphasizing the importance of a comprehensive and standardized approach to mitigate this critical oversight [9]. Furthermore, as recommended by Huchon et al. in their study, the implementation of a self-assessment questionnaire for gynecological patients could be beneficial [13]. This approach could be effectively incorporated into the triage documentation of all female patients of reproductive age.

The inherent limitations of this study stem from its small sample size and its circumscribed focus within a single NHS Trust. Consequently, the investigation serves to illuminate a broader spectrum of unanswered questions rather than provide definitive conclusions. The suboptimal outcomes observed in this audit likely reflect a systemic issue prevalent across multiple NHS Trusts. While this study reports no instances of mortality or EP, the small sample size precludes definitive conclusions regarding these critical outcomes. However, extrapolation of these findings to a larger population necessitates a sobering consideration: the potential for significant adverse consequences, including physical and psychological harm, miscarriage, and even mortality, cannot be dismissed.

## Conclusions

This audit, conducted within a single NHS Trust, revealed significant deficiencies in the initial gynecological assessment of female patients of reproductive age presenting with acute abdominal pain. Notably, documentation of crucial information, such as pregnancy status, menstrual history, and contraceptive use, was frequently inadequate. These findings suggest a potential systemic issue requiring broader attention within the NHS. To address this, a multi-pronged approach is necessary, encompassing targeted medical education, the implementation of standardized assessment protocols, and the routine electronic documentation of pregnancy status across all NHS Trusts. Continued research and data collection are crucial to fully understand the scope and impact of these deficiencies, ultimately informing the development of effective strategies to enhance patient safety and optimize clinical outcomes for women of reproductive age.
